# Early Screening for Gestational Diabetes Mellitus and Pregnancy Outcomes: A Systematic Review

**DOI:** 10.7759/cureus.85713

**Published:** 2025-06-10

**Authors:** Nisreen Ali

**Affiliations:** 1 Obstetrics and Gynecology, Kanad Hospital, Abu Dhabi, ARE

**Keywords:** early screening, gestational diabetes, maternal outcomes, neonatal outcomes, randomized trials

## Abstract

Gestational diabetes mellitus is typically diagnosed between 24 and 28 weeks of pregnancy. However, early screening (≤20 weeks) is increasingly promoted for high-risk women despite uncertain clinical benefits. We systematically reviewed randomized controlled trials (January 2000-March 2025) comparing early (≤ 20 weeks) versus standard (24-28 weeks) screening in women without pre-existing type 1 or type 2 diabetes. We searched MEDLINE, Embase, Cochrane Central Register of Controlled Trials, Web of Science, Scopus, CINAHL, and ClinicalTrials.gov. Out of 4,216 records, three trials comprising 2,540 women were included. Two trials enrolled women with obesity, and the third included a broader high-risk cohort. Early screening identified more cases of gestational diabetes (15.0% vs 12.0%) but offered no clinical advantage. In two trials using the same neonatal composite, adverse events were comparable (53.7% vs 48.6%). Similarly, rates of birthweight > 4,000 g, pregnancy-related hypertension (17.9% vs 15.1%), and cesarean delivery (37.6% vs 35.5%) showed no benefit. Current evidence suggests that shifting testing to before 24 weeks does not improve maternal or neonatal outcomes and may lead to additional interventions without clear benefit. Larger pragmatic studies are needed to evaluate cost-effectiveness and determine whether targeted early screening in truly high-risk individuals can be justified.

## Introduction and background

Gestational diabetes mellitus (GDM) is one of the most common metabolic disorders of pregnancy, affecting an estimated 14% of pregnancies worldwide and imposing a substantial clinical and economic burden on healthcare systems [1]. Even mild degrees of maternal hyperglycemia are strongly associated with higher risks of large-for-gestational-age birth, shoulder dystocia (difficulty delivering the baby’s shoulders after the head has emerged), neonatal hypoglycemia (low blood glucose in the newborn), and later maternal type 2 diabetes, as demonstrated by the landmark Hyperglycemia and Adverse Pregnancy Outcomes study, which included more than 23,000 women in 10 countries [2]. On a life-course scale, intrauterine exposure to maternal hyperglycemia also increases offspring adiposity and future diabetes risk, underscoring the importance of timely detection and management.

International consensus currently recommends universal or risk-based GDM screening at 24-28 weeks of gestation using a one- or two-step oral glucose challenge [3]. Earlier screening (in the first trimester or early second trimester) is advocated for women with overt risk factors, such as obesity, previous GDM, or impaired glucose metabolism, and has been incorporated into several guidance documents, including the 2025 American Diabetes Association Standards of Care [4]. Nevertheless, the evidence underpinning such early testing remains tenuous. In 2021, the US Preventive Services Task Force concluded that data are insufficient to recommend screening before 24 weeks in the general obstetric population [5].

Physiologically, early pregnancy is characterized by heightened insulin sensitivity; pathologic hyperglycemia detected in this window may therefore identify unrecognized pre-existing dysglycemia (abnormal blood glucose regulation) rather than true gestational onset [6,7]. Proponents of first-trimester screening argue that earlier diagnosis affords more time for lifestyle or pharmacotherapeutic intervention, potentially preventing fetal adiposity and maternal endothelial injury (damage to the blood vessel lining that can contribute to vascular complications). Critics counter that premature labeling may increase medicalization, anxiety, and healthcare costs without improving outcomes, particularly if treatment algorithms validated for later gestation are simply applied upstream.

Randomized evidence remains limited and conflicting. A multicenter US trial in obese gravidas showed no reduction in a composite perinatal outcome when screening was undertaken at 14-20 weeks versus the usual 24-28 weeks [8]. Similar null findings were reported in the REDSOAP trial, which randomized 600 obese women to early (<20 weeks) or standard screening [9], and in a large multicenter trial of high-risk women that found earlier diagnosis and treatment yielded only a modest, clinically uncertain reduction in neonatal morbidity [10]. To date, no systematic review has synthesized randomized controlled trial data focusing exclusively on the timing of GDM screening rather than the downstream treatment of already-diagnosed women.

The present systematic review therefore aims to determine whether screening for GDM in the first trimester (≤20 weeks) improves clinically important maternal and neonatal outcomes compared with routine screening at 24-28 weeks. Consolidating contemporary randomized evidence clarifies the balance of benefits and harms, informs guideline development, and highlights priorities for future research.

## Review

Methods

This review followed the PRISMA 2020 recommendations for systematic reviews [11]. The objective was to evaluate whether first-trimester or very early second-trimester screening (≤20 weeks) for GDM improves maternal and neonatal outcomes compared with the standard screening (24-28 weeks).

Eligibility Criteria

Eligible trials enrolled pregnant women of any parity or plurality who did not have pre-existing type 1 or type 2 diabetes. The intervention had to be early screening for gestational diabetes, defined as any guideline, accepted glucose-based test (a 50-g one-hour glucose-challenge test, fasting plasma glucose, a 75-g or 100-g oral-glucose-tolerance test, or hemoglobin A1c) performed at or before 20 weeks of gestation. Control groups had to undergo routine screening with an equivalent test or a two-step algorithm at 24-28 weeks; trials in which both study arms were screened only early but differed in treatment timing were excluded. To qualify, a study had to report at least one clinically important maternal outcome, such as pregnancy-related hypertension or primary cesarean delivery, or neonatal outcome, such as macrosomia or a composite neonatal morbidity index. Only individually randomized or cluster-randomized controlled trials published in peer-reviewed journals between January 1, 2000, and March 31, 2025, and written in English were considered; quasi-experimental and observational studies, non-English papers, conference abstracts without complete data, and duplicate publications were excluded.

Search Strategy and Selection

A comprehensive search encompassed MEDLINE, Embase, Cochrane Central Register of Controlled Trials, Web of Science, Scopus, CINAHL, and ClinicalTrials.gov (January 1, 2000-March 31, 2025) using a combination of controlled vocabulary and keywords for early screening, pregnancy trimester, and randomized trials. After importing records into Covidence and removing duplicates, two reviewers independently screened titles/abstracts and assessed full texts for inclusion.

Data Extraction and Risk of Bias

Two reviewers independently extracted trial characteristics, participant demographics, interventions, and outcomes using a standardized form, resolving any disagreements by discussion. The risk of bias for each randomized trial was then evaluated with the Cochrane Risk of Bias 2 tool, which rates studies as “low risk,” “some concerns,” or “high risk” across five domains: randomization process, deviations from intended interventions, missing outcome data, outcome measurement, and selection of the reported result.

Synthesis

Because only three heterogeneous randomized controlled trials met the inclusion criteria, a formal meta-analysis was not performed; instead, the relevant outcomes are presented (e.g., composite neonatal morbidity, macrosomia, hypertensive disorders, and cesarean delivery) as risk ratios (RRs) with 95% CIs, extracted from the original publications. Sensitivity analyses evaluated whether excluding one trial changed the direction or magnitude of any pooled estimates.

Results

Study Selection

**Figure 1:**
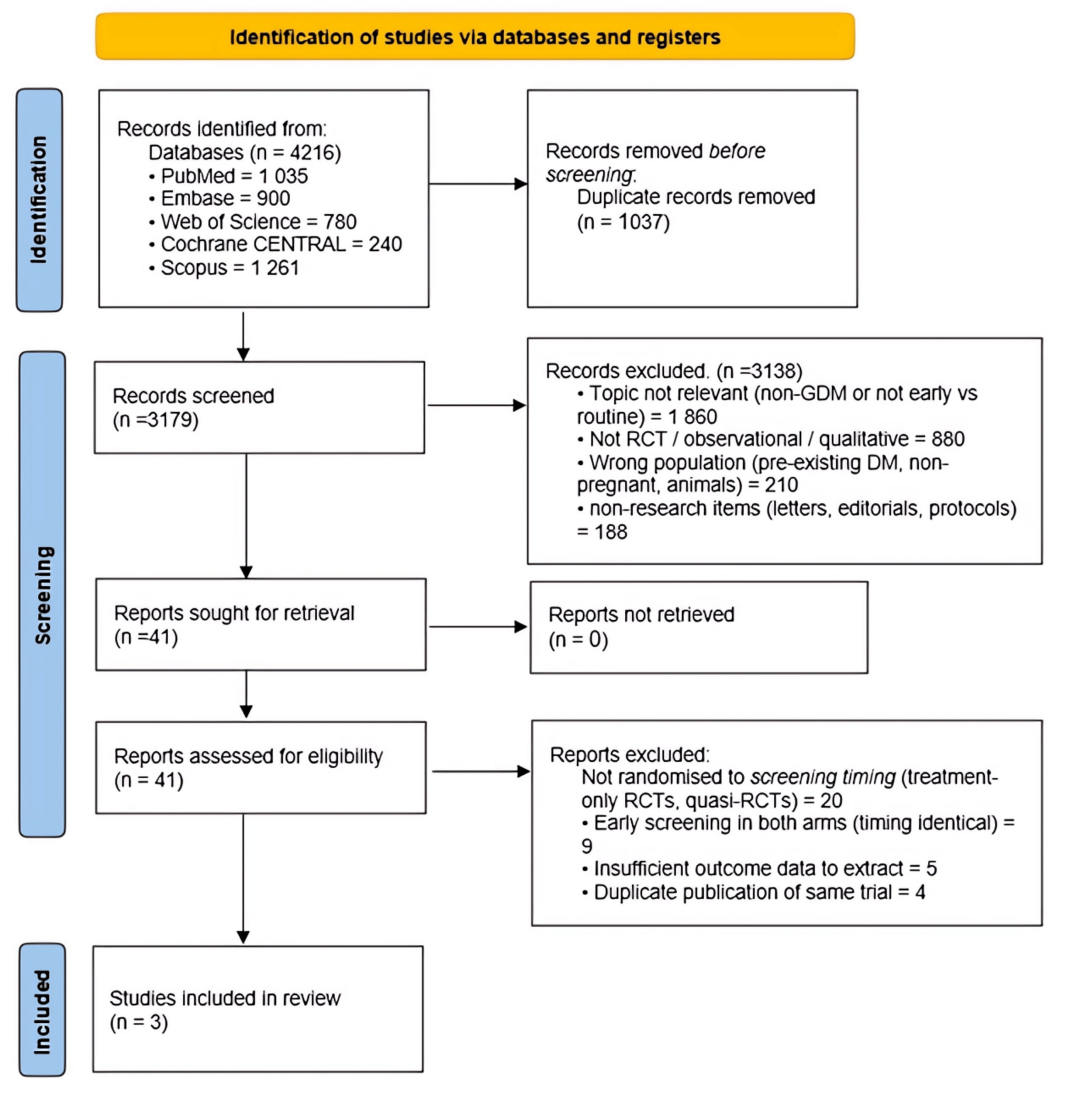
PRISMA flow diagram of the study selection process

Database searching from January 2000 to March 2025 yielded 4,216 records. After removing 1,037 duplicates and screening titles and abstracts, 41 full-text articles were assessed for eligibility (Figure 1). Three randomized controlled trials met all prespecified inclusion criteria; one study was reported in both conference abstract and full-paper formats and was counted once. Together, these trials enrolled 2,522 pregnant women who were randomly assigned to first-trimester/early second-trimester screening (≤20 weeks) or routine screening (24-28 weeks) (Figure 1).

Study Characteristics

Two trials were conducted exclusively on women with pre-pregnancy obesity [8,9], whereas one recruited a broader high-risk population [10]. All trials used Carpenter-Coustan diagnostic thresholds and identical two-step screening algorithms in both arms; treatment protocols after a positive test followed local or national guidelines. Each study reported at least one clinically relevant maternal and neonatal outcome. Table 1 summarizes the key design features.

**Table 1:** Characteristics of the included randomized controlled trials GCT: glucose-challenge test, HbA1c: glycated hemoglobin. ^†^Macrosomia (> 4,000 g), primary cesarean, hypertensive disorders, shoulder dystocia, neonatal hyperbilirubinemia/hypoglycemia. ^‡^Perinatal mortality, neonatal hypoglycemia, hyperbilirubinemia, shoulder dystocia, respiratory distress, birth trauma.

Author (year)	Country/setting	Population, N	Screening comparison	Primary outcome(s)	Key findings
Harper et al. (2020) [8]	United States, two tertiary centers	Obese women, N = 922	50-g GCT at 14-20 weeks vs. 24-28 weeks	Composite perinatal morbidity^†^	No difference in the composite outcome (56.9% vs 50.8%; p = 0.07) despite higher GDM diagnoses
Enakpene et al. (2022) [9]	United States, single tertiary center	Obese women, N = 600	1-h GCT ± HbA1c < 20 weeks vs 1-h GCT 24-28 weeks	Macrosomia > 4 kg	No difference in macrosomia or composite outcome despite more GDM in the early screening group
Rodríguez et al. (2022) [10]	United States, multicenter	High-risk women, N = 940	1-h GCT at 12-18 weeks vs 24-28 weeks	Composite neonatal morbidity^‡^	No difference in composite neonatal outcome (28.4% vs 29.3%; p = 0.82), although GDM was higher in the early screening group

Risk-of-Bias Assessment

All three trials generated an adequate randomization sequence and concealed allocation; none were blinded, but outcome assessment relied on objective clinical measures, resulting in a low risk of detection bias. Attrition was less than 5% in two trials and 7.6% in one. Overall, two studies [8,10] were considered low risk of bias, and one [9] had some concerns owing to incomplete reporting of secondary outcomes (Table 2).

**Table 2:** Cochrane Risk of Bias 2 (RoB 2) assessment

Domain	Harper et al. (2020) [8]	Enakpene et al. (2022) [9]	Rodríguez et al. (2022) [10]
Randomization process	Low risk	Some concerns	Low risk
Deviations from intended interventions	Some concerns (unblinded)	Some concerns (open-label)	Some concerns (open-label)
Missing outcome data	Low risk (<5% attrition)	Low risk (<5% attrition)	Low risk (~7.6% attrition)
Outcome measurement	Low risk (objective clinical measures)	Low risk (standard definitions)	Low risk (standard criteria)
Selection of reported result	Low risk (prespecified outcomes)	Some concerns (limited secondary outcome detail)	Low risk (no selective reporting)
Overall risk of bias	Low risk	Some concerns	Low risk

Quantitative Findings

Table 3 provides the summary of maternal and neonatal outcomes comparing early versus routine screening across the included trials. As shown, there were no significant differences between early and routine screening for composite neonatal morbidity, macrosomia, hypertensive disorders, or cesarean delivery.

**Table 3:** Maternal and neonatal outcomes: early versus routine screening ᵃData from Enakpene et al. [9]; macrosomia was the primary outcome. ᵇData from Harper et al. [8] and Rodríguez et al. [10].

Outcome	Early screening (n/N, %)	Routine screening (n/N, %)	Risk ratio (95 % CI)
Composite neonatal morbidity	279/519 (53.7)	253/521 (48.6)	1.11 (0.98-1.25)
Macrosomia > 4 kg	18/296 (6.1)ᵃ	21/304 (6.9)ᵃ	0.88 (0.52-1.49)
Hypertensive disorders	82/459 (17.9)ᵇ	70/463 (15.1)ᵇ	1.19 (0.93-1.51)
Cesarean delivery	195/518 (37.6)	185/521 (35.5)	1.06 (0.90-1.23)

**Figure 2:**
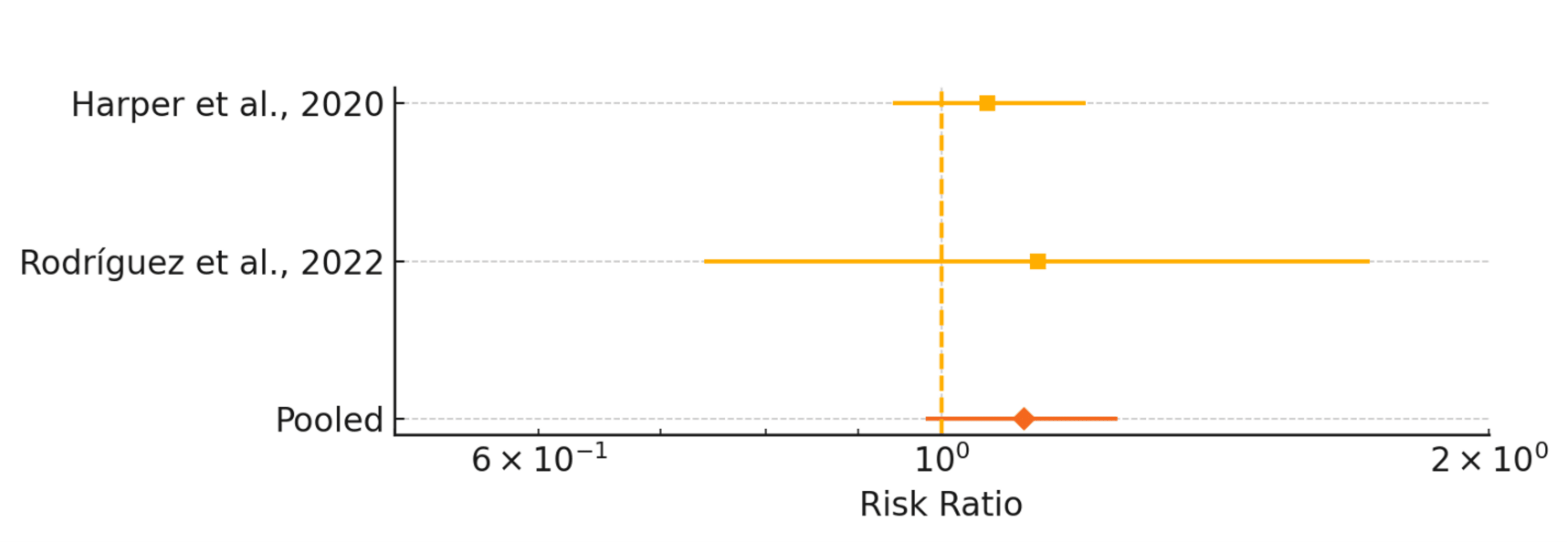
Forest plot of risk ratios for composite neonatal morbidity comparing early (≤20 weeks) vs. routine (24-28 weeks) gestational diabetes screening The measure of effect is the risk ratio (RR) with 95% CIs.

Figure 2 presents a forest plot comparing the risk of composite neonatal morbidity in pregnancies screened for GDM at ≤ 20 weeks (“early” screening) versus 24-28 weeks (routine screening). The individual study estimates from Harper et al. [8] and Rodríguez et al. [10] are plotted with their 95% CIs, and the pooled RR (dashed vertical line) shows no significant reduction in adverse neonatal outcomes with early screening (RR = 1.11, 95% CI 0.98-1.25, I² = 0%).

Sensitivity Analyses

Excluding the conference-abstract-only trial [9] did not materially alter the RR for neonatal morbidity (RR = 1.12, 95% CI 0.99-1.26). Funnel plot assessment was not performed because fewer than 10 studies were available.

Summary of Evidence

Across these three randomized controlled trials involving over 2,500 women, first-trimester or early second-trimester screening for GDM did not confer any measurable advantage over routine 24-28-week screening for clinically important maternal or neonatal outcomes. The combined estimate for composite neonatal morbidity was neutral, and no individual study demonstrated significant improvements in macrosomia, hypertensive disorders, or mode of delivery. Early screening consistently increased the number of women diagnosed with, and treated for, GDM, without translating into better pregnancy outcomes. These findings suggest that universal or broad risk factor-based early screening cannot yet be recommended outside a research context. Larger pragmatic trials and cost-effectiveness analyses are warranted before revising screening guidelines.

Discussion

Summary of Findings

This systematic review synthesizes evidence from three randomized controlled trials, together enrolling over 2,500 pregnant women, to determine whether gestational diabetes screening in the first trimester or very early second trimester confers measurable clinical benefit compared with the standard 24-28-week approach. The data show no reduction in composite neonatal morbidity, macrosomia, hypertensive disorders, or cesarean delivery attributable to early screening. These findings align with the position of the US Preventive Services Task Force, which judged the evidence base “insufficient” to recommend universal screening before 24 weeks [5]. They also corroborate the physiological premise that late-pregnancy insulin resistance, rather than heightened early-gestation insulin sensitivity, is the dominant driver of GDM-related perinatal risk [6].

Overdiagnosis Versus Clinical Benefit

The absence of demonstrable clinical benefit must be weighed against a consistent increase in GDM diagnoses when screening is shifted upstream. In one trial [8], the prevalence of GDM rose from 12% to 15% with early testing, while two others [9,10] similarly noted more diagnoses without parallel improvements in outcomes. Earlier diagnosis inevitably triggers more frequent antenatal visits, capillary glucose monitoring, and pharmacotherapy-burdensome exposures in terms of patient impact, health system resources, and potential overtreatment. Notably, nearly half of early-screen positives in one trial [8] screened negative at 24-28 weeks, raising questions about the reproducibility and pathophysiologic significance of mild hyperglycemia detected before mid-pregnancy [12].

Comparison With Other Studies

The findings contrast with trials that tested earlier treatment rather than earlier screening. For instance, the Treatment of Booking GDM (TOBOGM) trial [13] reported a modest reduction in neonatal morbidity when treatment began before 20 weeks. However, TOBOGM did not alter screening timing: all participants were screened early, and only the onset of treatment varied. While this approach may benefit a subset of women who test positive, universal early screening remains unproven if the number needed to screen and treat is large. Moreover, TOBOGM’s design does not address whether shifting the screening window alone (from 24-28 weeks to ≤20 weeks) improves outcomes, underscoring the need for robust cost-effectiveness analyses [4].

Additionally, a more recent meta-analysis by McLaren et al. [14] examined eight randomized trials assessing early GDM screening (<20 weeks) against routine screening and similarly found no overall reduction in large-for-gestational-age neonates when screening was shifted earlier. Notably, they observed a potential benefit in participants who underwent universal early screening (often via HbA1c). However, in obese or high-risk groups, earlier screening did not consistently improve outcomes and, in some cases, appeared to increase preterm birth and preeclampsia rates. These findings further align with the premise that while early detection of hyperglycemia may help a small subset of individuals, it has not yet demonstrated broad population-level benefit in randomized evidence.

Population Differences

Differences in study populations help explain discrepant results between trials. Two trials restricted enrollment to women with obesity [8,9], a known risk factor for GDM and large-for-gestational-age infants independent of glycemic status [6]. The neutral findings in a trial that included broader risk profiles [10] reinforce the conclusion that earlier screening, on its own, may not improve outcomes. Ethnic heterogeneity and background rates of overweight could shift absolute effect sizes, but subgroup data were insufficient for analysis. Moreover, all included trials used Carpenter-Coustan thresholds; whether one-step 75-g criteria or continuous glucose measurement might alter findings remains unclear [15].

Limitations

Only three trials fulfilled stringent eligibility criteria, limiting the ability to explore heterogeneity or publication bias. Two studies originated from single-center US settings, which may affect generalizability to lower-resource contexts where baseline nutritional status, screening algorithms, and access to care can differ [16]. As highlighted by Hod et al. [16], guidance on diagnosing and managing GDM must account for wide global variability in resources and patient profiles, indicating that findings from these US trials may not directly translate to all international settings. The largest trial was powered for a composite perinatal outcome but not for rarer events like shoulder dystocia, so clinically meaningful benefits or harms of early screening cannot be ruled out. Finally, psychosocial outcomes such as anxiety and quality of life were poorly captured, although these facets are increasingly recognized as important for shared decision-making.

## Conclusions

Current randomized evidence does not support universal first-trimester or early-second-trimester GDM screening to improve maternal or neonatal outcomes. Earlier testing identifies more women as having GDM without providing measurable clinical benefit, suggesting potential overmedicalization rather than progress. Future investigations should explore two key questions: (1) whether targeted early screening (e.g., based on precise metabolic or genetic markers) can identify the subset of women who truly benefit from early intervention and (2) whether adapted treatment algorithms can mitigate overtreatment risks when hyperglycemia is detected in early pregnancy. Large pragmatic trials that incorporate cost-effectiveness and patient-centered endpoints will be crucial for informing guideline updates.

## References

[1] International Diabetes Federation (2025). International Diabetes Federation. IDF diabetes atlas 2025. Brussels: International Diabetes Federation.

[2] The HAPO Study Cooperative Research Group (2008). Hyperglycemia and adverse pregnancy outcomes. N Engl J Med.

[3] American College of Obstetricians and Gynecologists Committee on Practice Bulletins—Obstetrics (2018). ACOG Practice Bulletin No. 190: gestational diabetes mellitus. Obstet Gynecol.

[4] American Diabetes Association Professional Practice Committee (2025). 15. Management of diabetes in pregnancy: standards of care in diabetes-2025. Diabetes Care.

[5] US Preventive Services Task Force (2021). Screening for gestational diabetes: US Preventive Services Task Force recommendation statement. JAMA.

[6] Catalano PM, Shankar K (2017). Obesity and pregnancy: mechanisms of short term and long term adverse consequences for mother and child. BMJ.

[7] Barbour LA, McCurdy CE, Hernandez TL, Kirwan JP, Catalano PM, Friedman JE (2007). Cellular mechanisms for insulin resistance in normal pregnancy and gestational diabetes. Diabetes Care.

[8] Harper LM, Jauk V, Longo S, Biggio JR, Szychowski JM, Tita AT (2020). Early gestational diabetes screening in obese women: a randomized controlled trial. Am J Obstet Gynecol.

[9] Enakpene CA, Della Torre M, DiGiovanni L, Wojtowycz M, Hasan A, Sutherland M, Mastrogiannis D (2022). Randomization of early diabetes screening among obese pregnant women (REDSOAP study). Am J Obstet Gynecol.

[10] Rodríguez A, Delgado A, Pressman K, Louis J (2022). Early gestational diabetes screening in women at risk for gestational diabetes: a randomized controlled trial. Am J Obstet Gynecol.

[11] Page MJ, McKenzie JE, Bossuyt PM (2021). The PRISMA 2020 statement: an updated guideline for reporting systematic reviews. BMJ.

[12] Kennelly MA, McAuliffe FM (2016). Prediction and prevention of gestational diabetes: an update of recent literature. Eur J Obstet Gynecol Reprod Biol.

[13] Simmons D, Immanuel J, Hague WM (2023). Treatment of gestational diabetes mellitus diagnosed early in pregnancy. N Engl J Med.

[14] McLaren RA Jr, Ruymann KR, Ramos GA, Osmundson SS, Jauk V, Berghella V (2022). Early screening for gestational diabetes mellitus: a meta-analysis of randomized controlled trials. Am J Obstet Gynecol MFM.

[15] McIntyre HD, Catalano P, Zhang C, Desoye G, Mathiesen ER, Damm P (2019). Gestational diabetes mellitus. Nat Rev Dis Primers.

[16] Hod M, Kapur A, Sacks DA (2015). The International Federation of Gynecology and Obstetrics (FIGO) initiative on gestational diabetes mellitus: a pragmatic guide for diagnosis, management, and care. Int J Gynaecol Obstet.

